# Quantitative Proteomics Shows Extensive Remodeling Induced by Nitrogen Limitation in *Prochlorococcus* *marinus* SS120

**DOI:** 10.1128/mSystems.00008-17

**Published:** 2017-05-30

**Authors:** Maria Agustina Domínguez-Martín, Guadalupe Gómez-Baena, Jesús Díez, Maria José López-Grueso, Robert J. Beynon, José Manuel García-Fernández

**Affiliations:** a Departamento de Bioquímica y Biología Molecular, Campus de Excelencia Agroalimentaria CEIA3, Universidad de Córdoba, Córdoba, Spain; b Centre for Proteome Research, Institute of Integrative Biology, University of Liverpool, Liverpool, United Kingdom; Marine Biological Laboratory

**Keywords:** marine cyanobacteria, nitrogen limitation, nitrogen metabolism, prochlorococcus, quantitative proteomics

## Abstract

*Prochlorococcus* requires the capability to accommodate to environmental changes in order to proliferate in oligotrophic oceans, in particular regarding nitrogen availability. A precise knowledge of the composition and changes in the proteome can yield fundamental insights into such a response. Here we report a detailed proteome analysis of the important model cyanobacterium *Prochlorococcus marinus* SS120 after treatment with azaserine, an inhibitor of ferredoxin-dependent glutamate synthase (GOGAT), to simulate extreme nitrogen starvation. In total, 1,072 proteins, corresponding to 57% of the theoretical proteome, were identified—the maximum proteome coverage obtained for any *Prochlorococcus* strain thus far. Spectral intensity, calibrated quantification by the Hi3 method, was obtained for 1,007 proteins. Statistically significant changes (*P* value of <0.05) were observed for 408 proteins, with the majority of proteins (92.4%) downregulated after 8 h of treatment. There was a strong decrease in ribosomal proteins upon azaserine addition, while many transporters were increased. The regulatory proteins P_II_ and PipX were decreased, and the global nitrogen regulator NtcA was upregulated. Furthermore, our data for *Prochlorococcus* indicate that NtcA also participates in the regulation of photosynthesis. *Prochlorococcus* responds to the lack of nitrogen by slowing down translation, while inducing photosynthetic cyclic electron flow and biosynthesis of proteins involved in nitrogen uptake and assimilation.

**IMPORTANCE** *Prochlorococcus* is the most abundant photosynthetic organism on Earth, contributing significantly to global primary production and playing a prominent role in biogeochemical cycles. Here we study the effects of extreme nitrogen limitation, a feature of the oligotrophic oceans inhabited by this organism. Quantitative proteomics allowed an accurate quantification of the *Prochlorococcus* proteome, finding three main responses to nitrogen limitation: upregulation of nitrogen assimilation-related proteins, including transporters; downregulation of ribosome proteins; and induction of the photosystem II cyclic electron flow. This suggests that nitrogen limitation affects a range of metabolic processes far wider than initially believed, with the ultimate goal of saving nitrogen and maximizing the nitrogen uptake and assimilation capabilities of the cell.

**Author Video**: An author video summary of this article is available.

## Abstract (precis)

*Prochlorococcus* is the most abundant photosynthetic organism on Earth, contributing significantly to global primary production and playing a prominent role in biogeochemical cycles. Here we study the effects of extreme nitrogen limitation, a feature of the oligotrophic oceans inhabited by this organism. Quantitative proteomics allowed an accurate quantification of the *Prochlorococcus* proteome, finding three main responses to nitrogen limitation: upregulation of nitrogen assimilation-related proteins, including transporters; downregulation of ribosome proteins; and induction of the photosystem II cyclic electron flow. This suggests that nitrogen limitation affects a range of metabolic processes far wider than initially believed, with the ultimate goal of saving nitrogen and maximizing the nitrogen uptake and assimilation capabilities of the cell.

## INTRODUCTION

The marine cyanobacterium *Prochlorococcus* (1, 2) is an important marine microbe model for ecological studies because of its abundance and significant contribution to global primary production (3). It maintains access to a very large pan-genome via horizontal gene transfer (4–6), and this genomic diversity has allowed *Prochlorococcus* to thrive over a broad range of environmental conditions (5, 7–12). Fifty-four *Prochlorococcus* genomes (13), representative of the different phylogenetic clades (14, 15) have been sequenced thus far. It is rarely feasible to use the genomic sequence of an organism to predict ecological function and environmental adaptation without complementary information regarding the proteins that are manifest under specific conditions. Fully sequenced microorganisms are attractive candidates for proteomic analysis, since these organisms allow rapid protein identification from the observed peptides by comparison with a list of protein sequences predicted from the genome. Therefore, quantitative proteomic analysis in *Prochlorococcus* has the potential to reveal mechanisms of adaptation to its environment in vast oceanic niches. There are not many physiological studies carried out with *Prochlorococcus*, due to the difficulties in culturing this microorganism (16), and consequently, there is a clear need for studies *in vivo* that address some important aspects of the physiology of *Prochlorococcus*.

Nitrogen, one of the main elements in life, is very scarce in the oligotrophic oceans inhabited by *Prochlorococcus* (17), the populations of which are limited by nitrogen but not phosphorus availability (18). In a previous study, we addressed the effect of nitrogen starvation on the redox proteome of *Prochlorococcus* sp. strain SS120, showing that this cyanobacterium responds with posttranslational redox changes to nitrogen starvation (19). Here we study the effect of azaserine, an inhibitor of glutamate synthase (20) on the proteome of the same *Prochlorococcus* strain. This inhibitor blocks the glutamine synthetase-glutamate synthase (GS-GOGAT) pathway, the main route for incorporation of ammonium ions (21), thus mimicking a situation of severe nitrogen starvation, and we used quantitative label-free proteomics to assess the effects on the *Prochlorococcus* SS120 proteome.

In terms of photophysiology, the SS120 strain represents an extreme within the *Prochlorococcus* genus because of its ability to grow at very low light levels (22). This strain is characterized by a nearly minimal gene complement for an oxyphototrophic organism (23). The compact genome of SS120 is maintained by selection and is related to the small cell volume of this organism (ca. 0.1 µm^3^), which is the theoretical lower limit for an oxyphototroph (24). Moreover, genome information can be correlated with fundamental characteristics of the genome and organism ecology in *Prochlorococcus marinus* SS120 (23). The genome is a single circular chromosome of 1,751,080 bp with an average G+C content of 36.4%. It contains 1,884 predicted protein-coding genes with an average size of 825 bp, a single rRNA operon, and 40 tRNA genes (23).

Cyanobacteria must sense and respond to carbon and nitrogen levels in order to adapt to changes in their source and availability, leading to an appropriate balance between carbon and nitrogen metabolism. This process involves diverse global regulators, including NtcA, P_II_, and PipX, to monitor the carbon/nitrogen balance by sensing the intracellular concentration of 2-oxoglutarate (2-OG) (25–29). Due to the absence of 2-OG dehydrogenase in *Prochlorococcus* (30), 2-OG can be metabolized only through the GS-GOGAT cycle, the central pathway of ammonium assimilation in cyanobacteria (31). Glutamine synthetase (GS) (EC 6.3.1.2) catalyzes the first step, the ATP-dependent synthesis of glutamine from glutamate and ammonium, and glutamate synthase (GOGAT) (EC 1.4.7.1) catalyzes the synthesis of glutamate from glutamine by the transfer of its amide group to the carbon skeleton 2-OG. Therefore, 2-OG is the final acceptor of the newly assimilated nitrogen and is produced by isocitrate dehydrogenase (ICDH) (EC 1.1.1.42). The concentration of 2-OG changes according to the nitrogen status of the cell (32). This confers a special importance to ICDH in the C/N balance.

Biochemical and physiological techniques have previously been used to define regulatory aspects of C/N metabolism among *Prochlorococcus* strains (16, 33–37). Global transcriptomics defined clear differences in the response of *Prochlorococcus* strain MED4 versus strain MIT9313 to the availability of different N sources (38), and there is some evidence for a response at the protein level (39). Recent advances in high-throughput techniques based on tandem mass spectrometry allow a greater insight concerning the integration, function, and regulation of the proteome (40). Here we used quantitative proteomics to study the effect of the addition of azaserine on the proteome of *Prochlorococcus marinus* SS120. The obtained results were further supported by quantitative reverse transcriptase PCR (qRT-PCR), measurement of the enzyme activities, and Western blotting for key enzymes such as ICDH and GS.

## RESULTS AND DISCUSSION

### Effect of azaserine addition on *Prochlorococcus* nitrogen and carbon metabolism.

**FIG 1:**
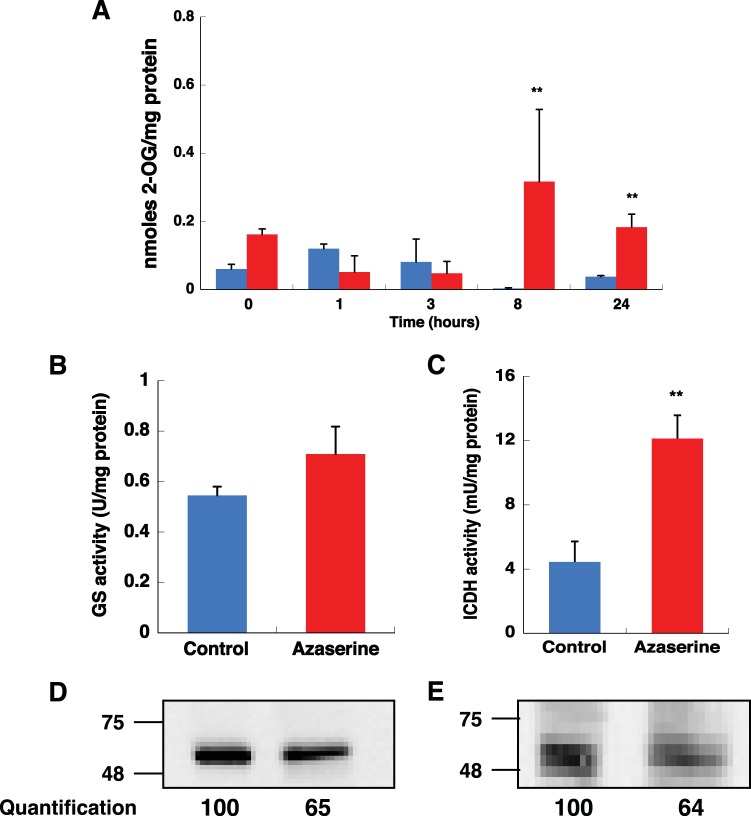
Effect of azaserine on cultures of *Prochlorococcus marinus* SS120. (A) Intracellular 2-OG concentration. Aliquots were taken at different times. The control culture (no addition) is shown by blue bars. Azaserine (100 µM) was added to cultures at time zero, and the values for these cultures are indicated by the red bars. (B) GS activity after 8-h treatment. (C) ICDH activity after 8-h treatment. The graphs represent the data from three independent biological replicates. Values are means plus standard deviation (error bars). Mean values that are significantly different (*P* ≤ 0.01) by Student’s *t* test are indicated by two asterisks. (D) Western blotting using anti-GS antibodies. (E) Western blotting using anti-ICDH antibodies. Densitometry results from the obtained bands are shown below the blots. One hundred percent corresponds to the intensity for the control situation. 75 and 48 indicate molecular mass in kilodaltons.

In *Prochlorococcus*, the only acceptor for newly assimilated nitrogen is 2-OG, forming glutamate via the GS/GOGAT pathway (30). 2-OG is the molecule responsible for the control of the C/N balance in cyanobacteria (21, 27, 29, 41, 42). Since azaserine is an inhibitor of GOGAT (20), its addition should increase the intracellular level of 2-OG. This hypothesis was confirmed in cultures of *Prochlorococcus marinus* SS120 (Fig. 1A). The addition of azaserine promoted a sharp increase in the intracellular concentration of 2-OG with a maximum peak at 8 h (*P* value of 0.0001 by Student’s *t* test) and remaining five times higher than the control condition even after 24 h.

We also focused our attention on the effects of azaserine on GS and ICDH as key enzymes of nitrogen metabolism. After 8 h of azaserine addition, GS activity increased ca. 25% in *Prochlorococcus* SS120 (Fig. 1B, *P* value of 0.0703 [not significant] by Student’s *t* test). This increment of enzymatic activity is the standard response shown by most photosynthetic organisms under nitrogen starvation (43). In contrast, GS protein concentration decreased after 8 h (Fig. 1D; 65.4% ± 20.6% with respect to the band intensity in control samples [100%]; *P* value of 0.0433 by Student’s *t* test). The addition of azaserine provoked a threefold, significant increase in ICDH activity after 8 h (Fig. 1C, *P* value of 0.0023 by Student’s *t* test), while a nonsignificant decrease of its concentration was found (Fig. 1E; 75.7% ± 16.2%; *P* value of 0.0602 by Student’s *t* test).

On the basis of the data in Fig. 1 and to investigate changes in the proteome associated with 2-OG accumulation, cultures were analyzed by proteomics 8 h after azaserine addition.

### Quantitative analysis of the *Prochlorococcus* SS120 proteome.

**FIG 2:**
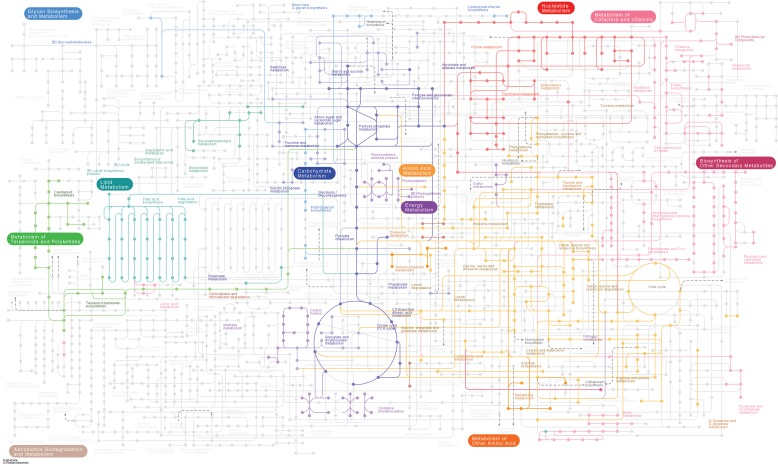
Proteome discovery. Metabolic diagram showing the pathways where the identified proteins are involved. We mapped KEGG identifiers for 396 proteins out of the 1,072 proteins identified. The diagram was made by using Pathview package in R software to highlight proteins with known function on top of the standard KEGG metabolic pathway scheme for *Prochlorococcus* SS120.

We identified 3,915 unique tryptic peptides in azaserine-treated cultures and 4,086 in control cultures at a false-discovery rate (FDR) of 5% at the peptide level. These peptides allowed the identification of 1,072 proteins with two or more unique peptides (see Table S1 in the supplemental material), 933 proteins common to both conditions, 81 unique to control samples, and 58 unique to azaserine-treated samples. The predicted number of proteins based on the genome is 1,884 for *Prochlorococcus* SS120 (23); hence, this value corresponds to 57% of the total proteome, which allowed us to characterize the most important metabolic pathways in the cell (Fig. 2). If we include proteins identified by a single peptide, the predicted proteome coverage increases up to 67.3% (1,269 proteins).

10.1128/mSystems.00008-17.7 TABLE S1  Proteins identified in both conditions, control and azaserine treatment conditions. Download TABLE S1, XLSX file, 0.1 MB. Copyright © 2017 Domínguez-Martín et al. 2017 Domínguez-Martín et al. This content is distributed under the terms of the Creative Commons Attribution 4.0 International license.

There are few global proteome studies in cyanobacteria (39, 40, 44–50). In a study on *Prochlorococcus marinus* MED4 (49), approximately 11% of the theoretical proteome was identified. Our study is the highest coverage of the proteome for any *Prochlorococcus* strain thus far. In a study carried out on *Nostoc punctiforme* ATCC 29133, 1,575 proteins were identified from a total of 7,432 predicted open reading frames (ORFs) (51, 52), which represents 21% of the proteome. Another proteomic study of *Synechocystis* sp. strain PCC 6803 identified 53% of the predicted proteome (48). The largest coverage described so far is a proteogenomic study in *Synechococcus* strain 7002, where 92% of the predicted protein-coding genes are identified (53).

Using String 10 software (http://string-db.org) (54) to analyze Kyoto Encyclopedia of Genes and Genomes (KEGG) pathways, we were able to map the identified proteins to 26 functional categories (Fig. S1). A BLAST search performed against the NCBI database to obtain functional descriptions of the hypothetical proteins (Table S2) suggested roles for 55 proteins, 26% of the total unknown.

10.1128/mSystems.00008-17.2 FIG S1  Enrichment analysis for KEGG pathways. Outline of the pathways obtained by using String v. 10 software. Download FIG S1, PDF file, 0.02 MB. Copyright © 2017 Domínguez-Martín et al. 2017 Domínguez-Martín et al. This content is distributed under the terms of the Creative Commons Attribution 4.0 International license.

10.1128/mSystems.00008-17.8 TABLE S2  Uncharacterized proteins identified and results from BLAST. Download TABLE S2, XLSX file, 0.1 MB. Copyright © 2017 Domínguez-Martín et al. 2017 Domínguez-Martín et al. This content is distributed under the terms of the Creative Commons Attribution 4.0 International license.

### Changes in the proteome of *Prochlorococcus* provoked by azaserine addition.

To evaluate changes in the proteome of *Prochlorococcus* SS120 after azaserine addition, we collected samples from three independent biological replicates after 8 h of growth in the absence of azaserine (control cultures) and in the presence of 100 µM azaserine.

**FIG 3:**
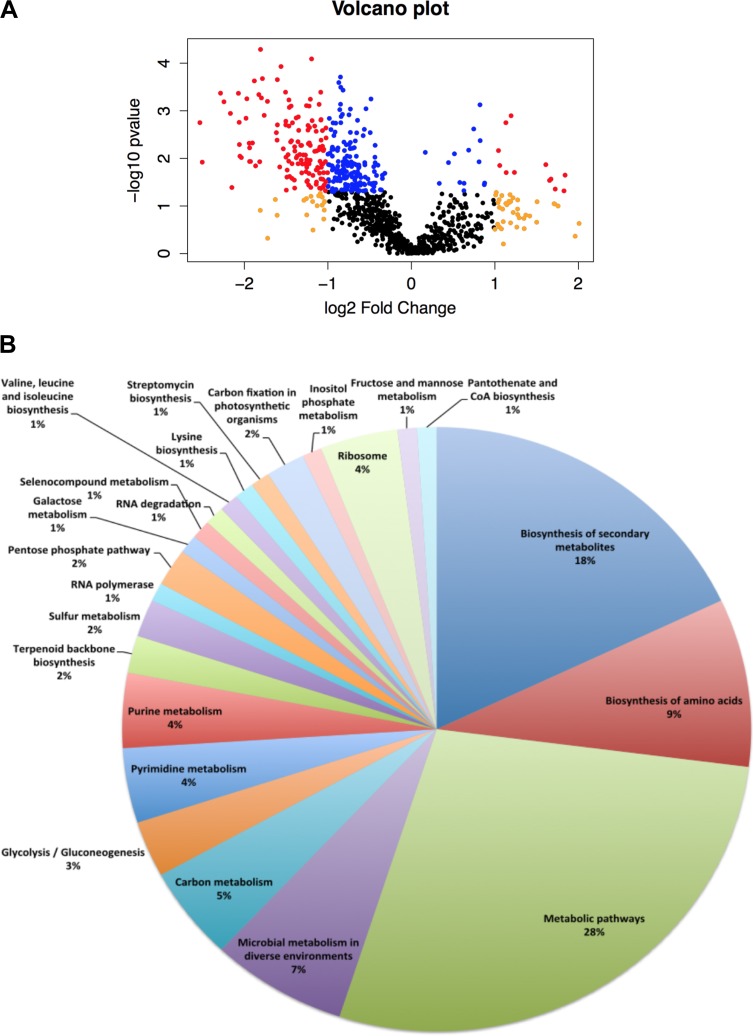
Analysis of the quantitative proteomic results. (A) Volcano plot showing the proteins. The proteins were indicated in color as follows: blue, fold change of <2, *P* value of <0.05; red, fold change of >2, *P* value of <0.05; yellow, fold change of >2, *P* value of >0.05. (B) Enrichment analysis. The 377 proteins significantly downregulated obtained by Progenesis were used for enrichment analysis in String 10 using default parameters. CoA, coenzyme A.

Label-free quantification was performed using Progenesis QI (Waters Corporation). Relative quantification revealed 408 proteins that were significantly altered with a *P* value of <0.05 and a *q* value (FDR) of <0.05 and using at least two unique peptides for quantification (Table S3). Results from the different biological replicates were comparable (Fig. S2). Principal-component analysis of the relative abundance of the proteins calculated by Progenesis QI (not shown) clustered the samples based on the treatment. Figure 3A shows a volcano plot highlighting the proportion of significant changes and the magnitude of those changes. Most proteins were downregulated (377 downregulated versus 31 upregulated), demonstrating that N starvation provokes a remarkable change on the proteome of *Prochlorococcus* SS120. Some main pathways related to carbon metabolism were downregulated (e.g., glycolysis; Fig. S3 and Table S4). The majority of upregulated proteins were involved in photosynthesis, being components of the photosystem II or proteins located in the thylakoid (Table 1 and Table S3).

10.1128/mSystems.00008-17.3 FIG S2  Exploratory analysis of the absolute quantification results. (A) Absolute proteome quantification. Correlation between the average quantification obtained per protein for control and azaserine-treated cultures. (B) Overlapping average quantification data obtained for each protein in control and azaserine-treated cultures. (C) Correlation plots using Pearson correlation. Download FIG S2, PDF file, 1.2 MB. Copyright © 2017 Domínguez-Martín et al. 2017 Domínguez-Martín et al. This content is distributed under the terms of the Creative Commons Attribution 4.0 International license.

10.1128/mSystems.00008-17.4 FIG S3  Changes in enzymes involved in pathways of glycolysis and gluconeogenesis. Outline of changes in the concentration of the indicated enzymes, represented by using Pathview on KEGG metabolic pathways. Download FIG S3, PDF file, 0.1 MB. Copyright © 2017 Domínguez-Martín et al. 2017 Domínguez-Martín et al. This content is distributed under the terms of the Creative Commons Attribution 4.0 International license.

10.1128/mSystems.00008-17.9 TABLE S3  Relative quantification (Progenesis). *P* value < 0.05, *k* value < 0.05. Two or more peptides were used for quantification. Absolute quantification of 1,007 proteins was identified under both conditions using Hi3 methodology. Download TABLE S3, XLSX file, 0.1 MB. Copyright © 2017 Domínguez-Martín et al. 2017 Domínguez-Martín et al. This content is distributed under the terms of the Creative Commons Attribution 4.0 International license.

10.1128/mSystems.00008-17.10 TABLE S4  Ribosomal and carbohydrate metabolism protein quantification. Download TABLE S4, XLSX file, 0.1 MB. Copyright © 2017 Domínguez-Martín et al. 2017 Domínguez-Martín et al. This content is distributed under the terms of the Creative Commons Attribution 4.0 International license.

Enrichment analysis of downregulated proteins (Fig. 3B) revealed pathways related to amino acid biosynthesis (isoleucine, valine, and leucine biosynthesis, FDRs of 1.81 × 10^−7^, 0.0316, and 0.0316, respectively). This response was expected due to the blocking of the main nitrogen assimilation pathway in the cell. Another downregulated pathway was nucleotide metabolism (pyrimidine and purine metabolism, FDRs of 0.00148 and 0.00197, respectively). The degradation of nonessential proteins and nucleotides is a well-established response to obtain nitrogen when cells are subjected to N limitation (55). Surprisingly, carbon metabolism (glycolysis and gluconeogenesis, galactose metabolism, carbon fixation, fructose and mannose metabolism, and the pentose phosphate pathways, with FDRs of 0.00134, 0.0109, 0.0316, 0.0442, and 0.00626; Table S4) was also downregulated, contrary to the response found in the alga *Phaeodactylum tricornutum* (56).

Absolute quantification using the Hi3 method was obtained for 1,006 proteins using at least two unique peptides for quantification, which corresponds to 53.4% of the predicted proteome of *Prochlorococcus* SS120 (Table S3).

### (i) Decrease in ribosomal protein concentration.

In order to assess whether translation was affected by azaserine, we evaluated the abundance of ribosomal proteins. Table S4 shows our results for all ribosome-related proteins. Based on our results, the average concentration of all proteins associated with the 30S or 50S subunit of the ribosome is 122.16 pmol/mg protein under control conditions, and 52.34 pmol/mg protein after azaserine addition—a 57% decrease in ribosomal protein concentration provoked by azaserine.

Furthermore, we assessed the azaserine effect on the ribosome concentration per cell, based on the ribosomal protein concentration. To this goal, we estimated that, under our conditions, 1 mg of total protein corresponds to 1.875 × 10^10^ cells, or 53 fg of protein per cell (see estimation in Text S1).

10.1128/mSystems.00008-17.1 TEXT S1  Estimation of ribosome concentration in *Prochlorococcus*. Download TEXT S1, DOCX file, 0.1 MB. Copyright © 2017 Domínguez-Martín et al. 2017 Domínguez-Martín et al. This content is distributed under the terms of the Creative Commons Attribution 4.0 International license.

The specific composition of the bacterial ribosomes has been suggested to change under different conditions (57), but it is generally accepted that most ribosomal proteins maintain a highly conserved stoichiometry at one copy per ribosome (58). If we assume this to be valid for *Prochlorococcus*, and make the corresponding calculations with the average ribosomal protein concentrations, we obtain the numbers of 3,900 ribosomes per cell under control conditions and 1,700 ribosomes per cell after azaserine addition. These results are in agreement with previous studies which estimated between 598 and 2,438 ribosomes per cell of *Prochlorococcus* (46). The decrease in the number of ribosomes after nutrient stress has also been observed in marine heterotrophic bacteria adapted to oligotrophic environments, as *Sphingomonas* sp. strain RB2256 (59). By committing a ribosome, there is a substantial amount of nitrogen that becomes available. This can be used, in turn, to provide nitrogen for other essential proteins, under conditions of strict N limitation. In this way, photosynthesis energy can be used to fuel protein degradation.

Ribosome concentration was also estimated in *Prochlorococcus* SS120, taking into account that cells from this strain have a volume of 0.144 µm^3^ (60). Our calculations gave the values of 27,000 ribosomes/µm^3^ under control conditions and 11,500 ribosomes/µm^3^ after azaserine addition. These values fall within the ranges reported for other organisms, such as *Escherichia coli* (6,200 to 65,500 ribosomes/µm^3^, depending on the growth rate [61]), *Sphingomonas* sp. RB2256 (4,000 to 40,000 ribosomes/µm^3^, depending on the nutrition state [59]), or *Rickettsia prowazekii* (17,000 ribosomes/µm^3^ [62]).

Our results suggest that one of the main responses to the blocking of nitrogen assimilation in *Prochlorococcus* is a pronounced decrease in the concentration of proteins involved in translation, particularly in the number of ribosomes per cell. This might contribute to save nitrogen resources (in a strategy similar to the dismantlement of phycobilisomes, reported in other cyanobacteria [63]). Further, translation would decrease the demand for amino acids. Both strategies are consistent with a physiological response of emergence under strict nitrogen limitation.

### (ii) Transporters are increased under azaserine addition.

Several upregulated proteins were membrane transporters: Q7VCK3 (porin homolog), Q7VA90 (phosphate-binding protein), Q7VA33 (Na^+^/proline symporter), and some ABC transporters (Table 1, Table S3, and Fig. S4). This family of ABC transporters drives uptake of ions, saccharides, lipids, and heavy metals across membranes (64).

10.1128/mSystems.00008-17.5 FIG S4  Effects of azaserine on the proteome of *Prochlorococcus* SS120 showing proteins that changed significantly after azaserine addition. Each panel shows the absolute quantification data obtained for proteins significantly changed (*P* value of <0.05): navy blue, control condition; light blue, azaserine treatment condition. The corresponding *P* value is shown in each panel. ATP6-PROMA, ATP synthase subunit α; PCBD_PROMA, divinyl chlorophyll *a*/*b* light-harvesting protein PcbD; PCBE_PROMA, divinyl chlorophyll *a*/*b* light-harvesting protein PcbE; PETD_PROMA, cytochrome *b*_6_-*f* complex subunit 4; PSBE_PROMA, cytochrome *b*_559_ subunit alpha; PSBF_PROMA, cytochrome *b*559 subunit beta; PSBL_PROMA, photosystem II reaction center protein L; Q7VA01_PROMA, GOGAT; Q7VA33_PROMA, Na^+^/proline symporter; Q7VA90_PROMA, phosphate-binding protein; Q7VAR2_PROMA, high-light-inducible protein Hli6; Q7VCK3_PROMA, porin homolog. Download FIG S4, PDF file, 0.04 MB. Copyright © 2017 Domínguez-Martín et al. 2017 Domínguez-Martín et al. This content is distributed under the terms of the Creative Commons Attribution 4.0 International license.

**TABLE 1:** List of selected groups of proteins belonging to different pathways showing relative and absolute quantification upon azaserine addition

Pathway and accession no. or protein	Description or name of protein	Total no. of peptides	No. of unique peptides	*P* value by ANOVA^a^	Max. fold change^b^	Highest^c^	Level of protein (pmol/mg) in:	
							Control culture	Azaserine-treated culture
Transporters								
Q7VCK3	Porin homolog	8	4	0.007	1.42	Azaserine	479 ± 44.75	685.62 ± 106.23
Q7VA90	Phosphate-binding protein	5	4	0.02	3.03	Azaserine	0.75 ± 0.02	62.17 ± 20.18
Q7VA33	Na^+^/proline symporter	3	3	0.03	1.26	Azaserine	1.75 ± 0.14	2.21 ± 0.32
Photosynthesis								
PSBF_PROMA	Cytochrome *b*_559_ subunit beta	2	2	0.001	2.2	Azaserine	35.63 ± 3.79	78.06 ± 19.65
PSBL_PROMA	Photosystem II reaction center protein L	2	2	0.02	3.6	Azaserine	9 ± 3.71	32.21 ± 19.54
PETD_PROMA	Cytochrome *b*_6_-*f* complex subunit 4	2	2	0.03	3.54	Azaserine	0.86 ± 0.24	3.06 ± 2.5
PSBE_PROMA	Cytochrome *b*_559_ subunit alpha	3	3	0.03	3	Azaserine	25.42 ± 7.11	80.97 ± 48.24
PCBE_PROMA	Divinyl chlorophyll *a*/*b* light-harvesting protein PcbE	11	10	0.04	2.97	Azaserine	100.26 ± 37.71	329.76 ± 251.22
PCBD_PROMA	Divinyl chlorophyll *a*/*b* light-harvesting protein PcbD	9	9	0.04	2.82	Azaserine	66.06 ± 22.82	176.47 ± 131.72
N metabolism								
Q7VDU1	NtcA	6	6	0.91	1.28	Azaserine	1.7 ± 0.27	2.29 ± 1.45
Q7VA51	P_II_	7	7	0.08	1.35	Control	217.7 ± 38	162.5 ± 49
Q7VDI6	PipX	3	3	0.06	1.6	Control	4.89 ± 0.91	3.14 ± 1.06
Q7VBQ4	GS	32	31	0.2	1.28	Control	653.17 ± 197.2	471.8 ± 111
Q7VA01	GOGAT	29	27	0.05	1.37	Azaserine	10.56 ± 2.49	15.12 ± 3.55
Q7V9S5	ICDH	12	11	0.63	1.14	Azaserine	3.84 ± 0.65	5.49 ± 1.42

a ANOVA, analysis of variance.

b Maximum fold change.

c Culture in which the protein reached its highest level.

An increase in porin abundance under P starvation was observed by Reistetter and coworkers (65). Porin PMM0709 from *Prochlorococcus* MED4, designated *phoE* by Martiny and colleagues (66), was strongly upregulated under P starvation and limitation (between 10- and 700-fold). PMM0709 shares the OprB domain (IPR007049) with two porins upregulated in our study, Q7VB31 and Q7VCK3, nominating them as members of the carbohydrate-selective porin OprB family. OprB was first identified in *Pseudomonas aeruginosa*. It facilitates the diffusion of a variety of compounds as glucose, glycerol, and fructose across the outer membrane (67). Under P limitation, PMM0709 may allow the transport of organic phosphorous compounds, such as sugar phosphates (66). We suggest that these upregulated proteins could allow the uptake of organic compounds such as glucosamine, galactosamine, *N*-acetylglucosamine, and *N*-acetylgalactosamine that can be used as N sources and are available in oligotrophic oceans (68).

### (iii) Photosynthetic cyclic electron transport is induced after azaserine addition.

**FIG 4:**
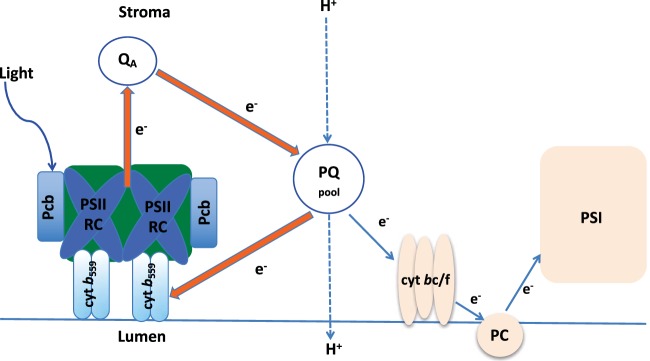
Outline of the cyclic electron flow around photosystem II in *Prochlorococcus* strain SS120. Orange arrows depict the cyclic electron flow, enhanced under N limitation. Blue arrows show the linear electron flow. Abbreviations: PQ, plastoquinones; PSII RC, photosystem II reaction center; cyt, cytochrome; PC, plastocyanine; PSI, photosystem I; Q_A_, A plastoquinone.

The upregulated proteins were mainly involved in photosynthesis (Table 1, Table S3, and Fig. S4). Therefore, we studied how the addition of azaserine affects the photosynthetic capacity in *Prochlorococcus*. The Fv′/Fm′ ratio decreased significantly (ca. 34%; *P* = 0.0001) after azaserine addition in spite of the maximum photosynthetic capacity (Fv/Fm) being affected considerably less (ca. 11%) (Fig. S5). This response was expected, given that azaserine blocks the incorporation of nitrogen into carbon skeletons, therefore preventing the utilization of the organic carbon compounds generated by photosynthesis. The fact that several photosynthetic proteins were upregulated, while photosynthetic efficiency decreases, suggests that cells are trying to obtain more light energy as a physiological response against N depletion. Since primary and secondary transport use metabolic energy, this response is consistent with the observed increase in the concentration of transporters described above. For instance, the ATP synthase subunit α (ATP6_PROMA) (Table S3 and Fig. S4) was ca. 2.3-fold higher in cells treated with azaserine. This enzyme is involved in energetic metabolism by generating ATP coupled to proton transport, thus playing an important physiological role when cells are exposed to environmental stress and require elevated ATP levels (69, 70). Blocking the GS-GOGAT pathway seems to stimulate the synthesis of ATP. Taking into consideration the upregulation of ABC transporters, which require ATP, we suggest that there is a link between these transporters that is actively working and the increased concentration of the ATPase in order to generate sufficient energy to promote uptake of nutrient compounds. In fact, all proteins with increased abundance (Table 1) are involved in cyclic electron transport around photosystem II. These proteins include photosystem II reaction center L protein, subunit 4 of cytochrome *b*_6_*f*, and the antenna proteins encoded by the *pcbD* and *pcbE* genes in *Prochlorococcus* SS120 (71). Furthermore, the concentrations of α and β subunits of cytochrome *b*_559_ were also increased (Table 1). This complex is required for the functioning of photosystem II, but it does not participate in the linear electron transport chain: it seems to be involved in cyclic electron transport around photosystem II (72), allowing the synthesis of ATP even if CO_2_ cannot be incorporated due to the blocking of N assimilation. Figure 4 outlines the process of cyclic electron transport in this *Prochlorococcus* strain, showing the proteins mentioned above.

10.1128/mSystems.00008-17.6 FIG S5  Effect of azaserine on the photosynthetic capacity of *Prochlorococcus* SS120. Azaserine (100 µM) was added to the cultures, and the cells were collected after 8 h. (A) Maximum photosynthetic capacity; (B) photosynthetic capacity. Download FIG S5, PDF file, 0.01 MB. Copyright © 2017 Domínguez-Martín et al. 2017 Domínguez-Martín et al. This content is distributed under the terms of the Creative Commons Attribution 4.0 International license.

NtcA promoters are present in the *Prochlorococcus* genomes, including strain SS120, for a variety of genes involved in various stages of the photosynthesis and carbon fixation processes (73). This strongly suggests that those genes are regulated by NtcA. In fact, it has been shown that nitrogen assimilation and photosynthesis are highly orchestrated processes (74). Nitrogen assimilation is linked to photosynthesis, since the uptake of nitrogen-containing compounds is powered by ATP and reducing power is generated by photophosphorylation (75). A high level of 2-OG (high C/N ratio) might be an indication of a relatively strong photosynthetic activity compared to the ongoing level of N assimilation.

On the other hand, the intensity of photosynthesis depends on the availability of N in the environment: low levels of N availability are associated with low levels of photosynthesis (Fig. S4). N deprivation depresses photosynthesis by inducing degradation of the photosynthetic apparatus (chlorosis) (76). Interestingly, when there is N available, photosynthesis resumes rapidly (76, 77). These facts strongly indicate that the photosynthetic process is able to sense the availability of nitrogen, and accordingly adapt its activity.

Our experimental conditions promoted an increment in 2-OG due to the blocking of the main nitrogen assimilation pathway. 2-OG binding to NtcA promoted the transcription of genes related to N metabolism. Besides, *in silico* putative binding sites for NtcA for many genes involved in various stages of photosynthesis have been reported, as for instance chlorophyll *a*/*b*-binding proteins (73). Interestingly, we found two divinyl chlorophyll *a*/*b* light-harvesting proteins (PcbD and PcbE) upregulated 2.80- and 2.9-fold, respectively (Fig. S4). Another example is the ferredoxin-NADP oxidoreductase (Q7VBH1), encoded by *petH*, which was 1.6-fold higher under azaserine treatment. This enzyme is involved in the last step of oxygenic photosynthesis and provides NADPH for anabolic reactions. Further, NtcA activates transcription of the *petH* promoter in *Synechocystis* sp. PCC 6803 (78). This suggests that NtcA plays an important role in coordinating the activities of the N assimilation and photosynthesis processes by currently unknown mechanisms.

It is worth noting that another photosynthesis-related protein was significantly upregulated, the high-light-inducible protein Hli6 (Q7VAR2 [Fig. S4]). In the proteome of *Prochlorococcus* SS120, there are 14 Hli proteins, due to very recent gene duplication events. These small polypeptides (35 to 150 amino acids long) are important for survival of cyanobacteria during exposure to high light (79). Hli polypeptides also accumulated under other stress conditions (N and S limitation, cold stress [80]). Under these conditions, Hli proteins help cells to absorb the excess excitation energy which causes hyperreduction of the acceptor side components of photosystem II, formation of triplet chlorophyll, generation of singlet oxygen within antenna and the reaction centers, and production of superoxide radicals (80, 81).

### (iv) Effects of azaserine on proteins related to N metabolism.

**FIG 5:**
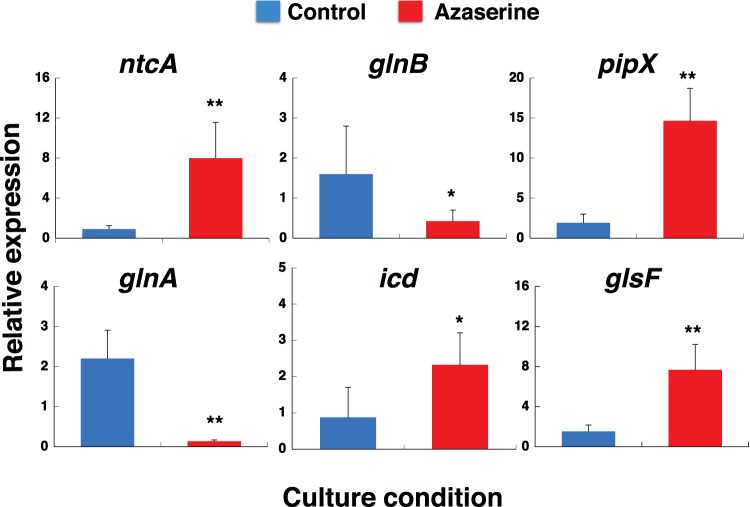
Effect of azaserine addition on gene expression in *Prochlorococcus* SS120. Azaserine (100 µM) was added to cultures. Cells were collected after 8 h, and gene expression was measured by qRT-PCR. Data are the average values for six independent biological replicates. Error bars correspond to standard deviations. Mean values that are significantly different by Student’s *t* test are indicated by asterisks as follows: *, *P* ≤ 0.05; **, *P* ≤ 0.01.

The regulatory system composed of NtcA (UniProt accession no. Q7VDU1), P_II_ (UniProt accession no. Q7VA51), and PipX (UniProt accession no. Q7VDI6) was also identified in our proteomic analysis. P_II_ and PipX were downregulated (Table 1). In contrast, NtcA concentration increased after azaserine treatment. We expected the NtcA and PipX proteins to be upregulated after azaserine addition. Therefore, we decided to determine their gene expression under the same conditions (Fig. 5). The expression of *ntcA* was upregulated in good agreement with the results at the protein level. However, the regulatory protein PipX was downregulated at the protein level, but *pipX* expression was upregulated. The difference between the gene expression level and the protein expression level could be due to the lag between transcription and translation described in *Prochlorococcus* (46). It could also be due to a delayed response of PipX with respect to NtcA, since it enhances the transcription of genes after prolonged stress (82). These results fit nicely with the model described by Espinosa and coworkers (82). The same good correlation occurred between the expression level and the protein content for *glnB* (P_II_ protein), *glsF* (GOGAT), *icd* (ICDH), and *glnA* (GS) 8 h after the addition of azaserine (Fig. 5).

A recent study on the global metaproteome in the ocean focused on the identification of cyanobacterial nutrient stress biomarkers using natural samples (83). In this study, Saito and coworkers (83) showed that proteomic biomarkers could diagnose ocean metabolism and demonstrated that *Prochlorococcus* actively and simultaneously deploys multiple biochemical strategies to cope with low-nutrient conditions in the oceans. Interestingly, one of the proteins described as biomarkers was the transcription factor NtcA. This is in good agreement with the main role of NtcA as the master regulator controlling the transcription of genes related to nitrogen metabolism in cyanobacteria (84). The distribution of NtcA binding sites in the cyanobacterial genome is much wider than has been previously thought (85). In *Anabaena* sp. strain PCC 7120, eight functional categories were identified as NtcA targets after 3 h of N deficiency. These categories are N metabolism and N fixation-related, regulatory functions, photosynthesis, respiration, transport and binding proteins, among others (85). The results of our study are consistent with those results and suggest that NtcA is also responsible for the control of a wide range of pathways in *Prochlorococcus*.

### Conclusions.

The goal of this study was to gain a global proteomic perspective on the adaptability of the cyanobacterium *Prochlorococcus* SS120 to N deprivation. For that purpose, we used a specific inhibitor of GOGAT, azaserine, to mimic the effect of N deprivation in the cell. The relationship between azaserine treatment and the increment in 2-OG concentration was demonstrated (Fig. 1), and then, the effect on the proteome was analyzed in detail. We obtained 57% coverage of the predicted proteome (Fig. 2), the highest coverage reported for a *Prochlorococcus* strain.

Our results strongly suggest that NtcA not only controls N metabolism but also photosynthesis in response to N stress in *Prochlorococcus*, as has been demonstrated in the open ocean (83) and suggested by bioinformatic studies (73) in other cyanobacteria.

*Prochlorococcus* SS120 responds to N deprivation by diminishing the majority of the biosynthetic metabolism pathways but increases the abundance of proteins involved in N uptake and assimilation. Further, the photosystem II cyclic electron transport was increased as a source of ATP production. In turn, this ATP could be used in the active uptake of other forms of N, as amino sugars (68).

## MATERIALS AND METHODS

### *Prochlorococcus* strains and culture conditions.

*Prochlorococcus marinus* strain SS120 (low-light-adapted ecotype) was routinely cultured in polycarbonate flasks (Nalgene) using PCR-S11 medium as described previously (16). The seawater used as basis for this medium was kindly provided by the Instituto Español de Oceanografía (Spain). Cultures were grown in a culture room at 24°C under continuous blue irradiance (4 µE m^−2^ s^−1^). Growth was determined by measuring the absorbance of cultures at 674 nm, and cells were collected during the exponential phase of growth.

### Cell collection.

Cultures (10 liters) reaching 0.05 units of absorbance at 674 nm were split into two aliquots: one was used as the control culture, while 100 µM azaserine [*O*-(2-diazoacetyl)-l-serine; Santa Cruz Biotechnology, CA] was added to the other. They were then kept under standard light and temperature conditions, and the cells were collected at the indicated times. The cells were harvested at 26,000 × *g* for 8 min at 4°C using an Avanti J-25 Beckman centrifuge equipped with a JA-14 rotor. After pouring out most of the supernatant and carefully pipetting out the remaining medium, the pellet was directly resuspended in cold Tris-HCl (50 mM) (pH 7.5) at a proportion of 1 ml of buffer per liter of culture. For protein assays and RNA analysis, the pellet was resuspended in 10 mM sodium acetate (pH 4.5) supplemented with 200 mM sucrose and 5 mM EDTA. For proteomic studies, cells were resuspended in 2 ml of cold 25 mM ammonium bicarbonate and 1 mM protease inhibitor cocktail (Sigma). Samples were stored at −20°C or −80°C until used.

### Preparation of cell extracts.

To determine the 2-OG concentration, cell extracts were obtained by centrifuging the thawed extracts for 10 min at 16,900 × *g* at 4°C.

For enzymatic assays, Western blotting analyses, and proteomic studies, cells were broken in a French pressure cell (SLM/Aminco model FA-079) at 16,000 lb/in^2^. The obtained extracts were centrifuged for 10 min at 16,900 × *g* at 4°C.

### Protein concentration.

Protein concentration was determined using the Bio-Rad protein assay kit, based on the Bradford method (86), following the instructions of the manufacturer.

### In-solution trypsin digestion of protein extracts.

Samples containing 100 µg of protein were incubated with RapiGest (Waters Corporation) at a final concentration of 0.05% (wt/vol) for 10 min at 80°C. Samples were then reduced with 3 mM dithiothreitol (DTT) for 10 min at 60°C, followed by alkylation with 9 mM iodoacetamide (IAM) for 30 min in the dark at room temperature. Finally, trypsin (50:1) was added, and samples were incubated overnight at 37°C. To stop the proteolytic reaction and to inactivate and precipitate the detergent, trifluoroacetic acid (TFA) (final concentration, 0.5% [vol/vol]) was added, followed by incubation for 45 min at 37°C. To remove all insoluble material, samples were centrifuged at 13,000 × *g* for 15 min at room temperature. Completeness of digestion was checked by SDS-PAGE (not shown).

### LC-MS/MS.

All samples were analyzed as tryptic peptides, resolved by high-resolution liquid chromatography (LC) (U3000 Thermo Scientific) prior to tandem mass spectrometry (MS/MS). The Q Exactive (Thermo Scientific) system was operated in data-dependent acquisition mode. The peptide mixture was trapped onto a Symmetry C_18_ precolumn (180-µm inner diameter [i.d.]; 20 mm long; 5-µm particles) (Waters Corporation) over 3 min, at a flow rate of 25 µl/min in 2% (vol/vol) acetonitrile–0.1% (vol/vol) formic acid. Bound peptides were resolved on a NanoAcquity ultrahigh-performance liquid chromatography C_18_ column (75-µm i.d.; 150 mm long; 3-µm particles) (Waters Corporation) at 300 nl/min over a 240-min linear gradient from 3 to 85% (vol/vol) acetonitrile in 0.1% (vol/vol) formic acid, controlled by IntelliFlow technology. The 10 most intense multiply charged ions were isolated and sequentially fragmented. Precursors selected were dynamically excluded for 20 s.

### Proteomic data analysis.

Peak lists were generated by Proteome Discoverer 1.4 (Thermo Scientific) using default parameters. The peak lists obtained were searched against a database composed of all entries in the UniProt database (http://www.uniprot.org) for *Prochlorococcus* strain SS120 (retrieved on 18 May 2015; 1,881 entries) using MASCOT as the search engine (version 2.4.0; Matrix Science, Inc.). The *Prochlorococcus* SS120 genome annotation contains 665 (35.3% of the proteome) hypothetical proteins, of which we identified 212. Carbamidomethylation was set as a fixed modification, and methionine oxidation was set as a variable modification, allowing one trypsin missed cleavage, a mass tolerance of 10 ppm for precursors and 0.01 Da for fragment ions. The false-discovery rate (FDR) was calculated using the decoy database option in MASCOT. LC-MS/MS data were processed for label-free quantification using Progenesis QI (Waters Corporation). As an internal standard for Hi3 absolute quantification, 50 fmol of rabbit phosphorylase B (UniProt accession no. P00489) (MassPREP digestion standard; Waters Corporation) was added to the sample to allow intensity-based proteomics to be converted to absolute quantification using Hi3 in Progenesis QI (87). Similar proteins were grouped, and only nonconflictive features (unique peptides) were used for quantification. For a protein to be considered significantly differentially expressed, it has to be identified and quantified using at least two unique peptides and has a *P* value of ≤0.05 and a *q* value of ≤0.05.

### Enzymatic assays.

Glutamine synthetase transferase activity was determined as previously described (88), during 30 min at 37°C. The reaction mixture contained 100 mM glutamine, 10 mM sodium hydroxylamine, 50 µM manganese chloride, 10 µM ADP, and 50 mM sodium arsenate in 0.2 M morpholinepropanesulfonic acid (MOPS) (pH 7.0) for 30 min at 37°C. Isocitrate dehydrogenase activity was determined as previously described (89) with modifications (33). The reaction mixture contained 840 µl of 50 mM Tris (pH 7.5), 20 µl of 100 mM MnSO_4_, 20 µl of 10 mM NADP^+^, 20 µl of 100 mM d,l-isocitrate, and 100 µl of cell extract. Isocitrate was added last to start the reaction. NADPH production was monitored by determining absorbance at 340 nm for 10 min in quartz cuvettes with the temperature set at 40°C. For all enzymatic activities, one unit of activity is the amount of the enzyme that transforms 1 µmol of substrate per min.

### Determination of the intracellular concentration of 2-OG.

An enzymatic method based on the oxidation of NADPH in the reaction catalyzed by the glutamate dehydrogenase (GDH) was used (33). The reaction mixture contained 85 mM Tris-HCl (pH 8.0), 0.2 mM NADPH, 5 µg (ca. 0.15 IU) of glutamate dehydrogenase enzyme (Fluka), 100 mM NH_4_Cl, and 200 µl of cell extract from *Prochlorococcus* (the total volume of the enzymatic mixture was 1 ml). The NADPH consumption was monitored by measuring the absorbance at 340 nm for 10 min in quartz cuvettes with the temperature set at 35°C.

### Detection of ICDH and GS by Western blotting.

The Western blotting procedure was performed as described in detail elsewhere for ICDH (33) and GS (90). Crude extracts from *Prochlorococcus* were prepared as described above. Fifteen micrograms of protein was loaded in each lane, subjected to SDS-PAGE, and transferred to a nitrocellulose membrane. After the membrane was blocked, it was incubated overnight with primary antibody (either anti-glutamine synthetase or anti-isocitrate dehydrogenase from *Synechocystis* sp. PCC 6803, kindly provided by M. I. Muro-Pastor and F. J. Florencio) at the appropriate dilution in Tris-buffered saline with Tween 20 (TBS-T) and 1% bovine serum albumin at 4°C with gentle shaking. The membrane was then washed three times for 15 min each time with TBS-T buffer. The membrane was incubated with a secondary antibody (anti-immunoglobulin from rabbit; labeled with peroxidase; Sigma) diluted 1:2,000 (vol/vol) in TBS-T for 30 min at room temperature with gentle shaking and washed three times for 15 min each time with TBS-T buffer. The immunoreacting material was detected by using the ECL Plus Western blotting detection system (General Electric Healthcare), according to the manufacturer’s instructions. Chemiluminescent signal was detected using a LAS-3000 camera (Fujifilm). Densitometric quantification of the Western blotting bands was performed by using the Quantity One software from Bio-Rad.

### qRT-PCR analysis of gene expression.

We followed the procedure described previously in detail (90). RNA was isolated from 500-ml culture samples by using TRIsure RNA isolation reagent (Bioline) following the manufacturer’s recommendations, with the addition of 133 µl of 8 M LiCl, an additional precipitation step included at the end of the procedure to improve RNA quality. RNA was treated with RNase-free DNase I (Ambion) following the manufacturer’s instructions, and the absence of contaminating genomic DNA was assessed using a PCR test. Synthesis of cDNA by the reverse transcriptase (RT) reaction from the RNA samples was carried out using the iScript cDNA synthesis kit (Quanta). One microgram of RNA was reverse transcribed in a reaction mixture with a total volume of 20 µl.

Real-time quantitative PCRs were performed in triplicate using SsoFast Eva Green SuperMix from Bio-Rad. An iCycler IQ multicolor real-time PCR detection system from Bio-Rad was used for quantitative detection of amplified PCR products using the following thermal cycling conditions: (i) 95°C for 2 min and (ii) 50 cycles, with 1 cycle consisting of 95°C for 15 s, followed by 58°C for 30 s and 72°C for 30 s. At the end, reactions were checked to discard false amplifications by verifying the melting points of the PCR products and determining the fluorescence between 65 and 100°C, with increases of 0.5°C measured each 10 s.

The sequences of the primers used are described elsewhere (37). The sequences for the *pipX* gene were RT-FPS: 5′-CCACTTTTGGGATGCTTTAT-3′ and RT-RPS: 5′-ACTTCAAAATCTGCACCTCG-3′. The relative change in gene expression was endogenously normalized to that of the *rnpB* gene, encoding RNase P, calculated using the 2^−ΔΔCt^ method (91). No change in *rnpB* expression was observed under our experimental conditions.

### Determination of effective photochemical quantum yield.

Culture samples (250 ml) were centrifuged at 26,000 × *g* for 8 min at 4°C. The pellet was resuspended in 2 ml of PCR-S11 medium and placed in a 24-well culture plate (Biofil). Cells were dark adapted for 30 min prior determination of Fo and Fm. The fluorescence of the chlorophyll was then measured using a system imaging-PAM WALZ IMAG-K5 (Heinz Walz GmbH, Effeltrich, Germany). The photosynthetic radiations used were 11 and 26 µE m^−2 ^⋅^ ^s^−1^.

### Statistical analysis.

Experiments were carried out with at least three independent biological samples. The results are shown with error bars corresponding to the standard deviations. Significance of data was assessed by using Student’s *t* test, and indicated in figures with asterisks as follows: *, *P* ≤ 0.05; **, *P* ≤ 0.01. Data were analyzed and visualized using Aabel package (Gigawiz Ltd. Co.) and R (v.3.2) (92). KEGG pathway visualization was performed using Pathview package from Bioconductor (93). Protein-protein interactions were analyzed with the STRING v. 10 software (54), available at http://string.embl.de/, by using the default parameters.

### Data availability.

The mass spectrometry proteomic data have been deposited to the ProteomeXchange Consortium via the PRIDE (94) partner repository with the data set identifier PXD005745.
