# Supplementary figures and images for: Quantitative Proteomics Shows Extensive Remodeling Induced by Nitrogen Limitation in Prochlorococcus marinus SS120
Source: mSystems. 2017 May 30;2(3):e00008-17. doi: 10.1128/mSystems.00008-17 (PMC5451487; doi:10.1128/mSystems.00008-17)

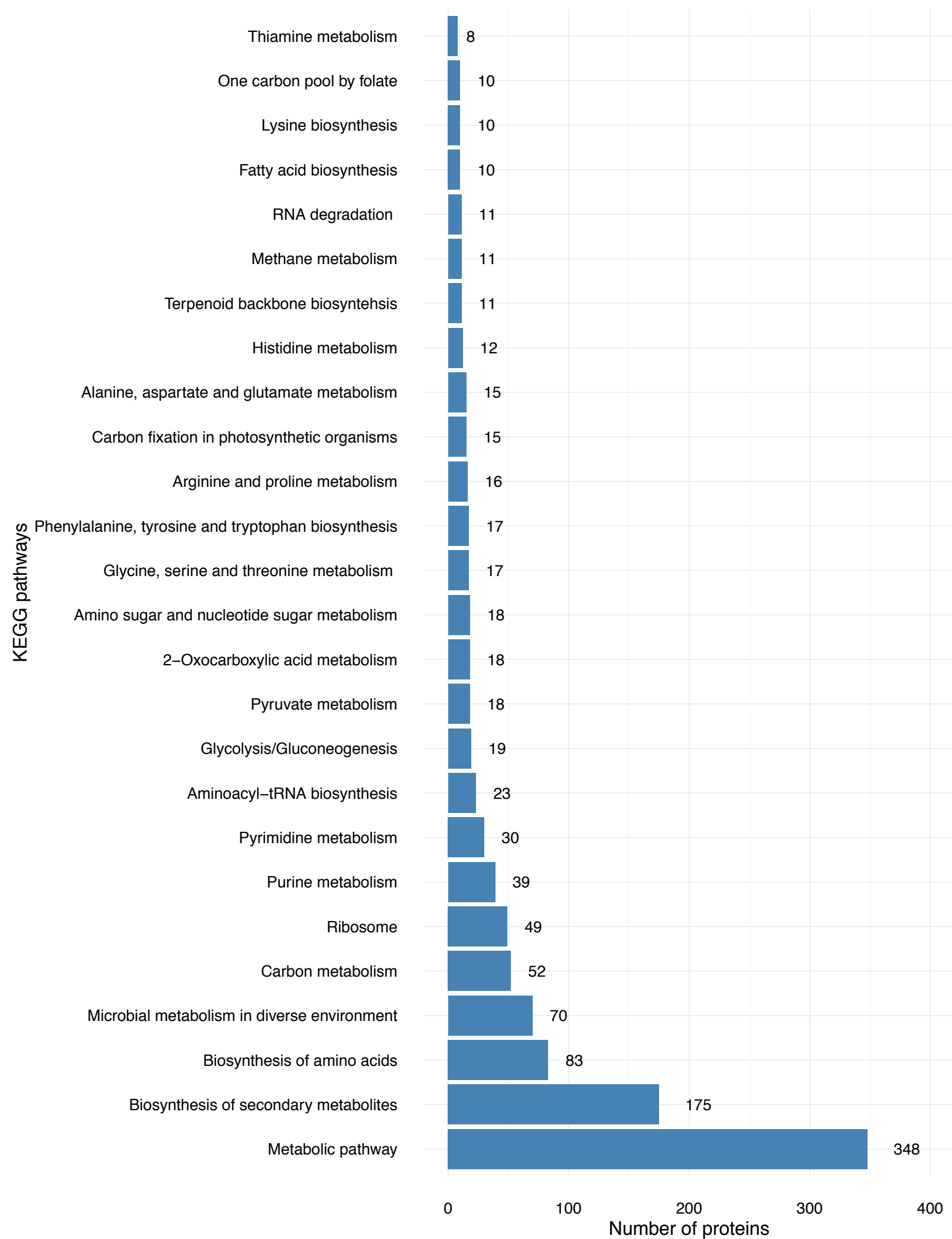

Supplement: FIG S1 [file sys003172107sf1.pdf]

# GLYCOLYSIS / GLUCONEOGENESIS

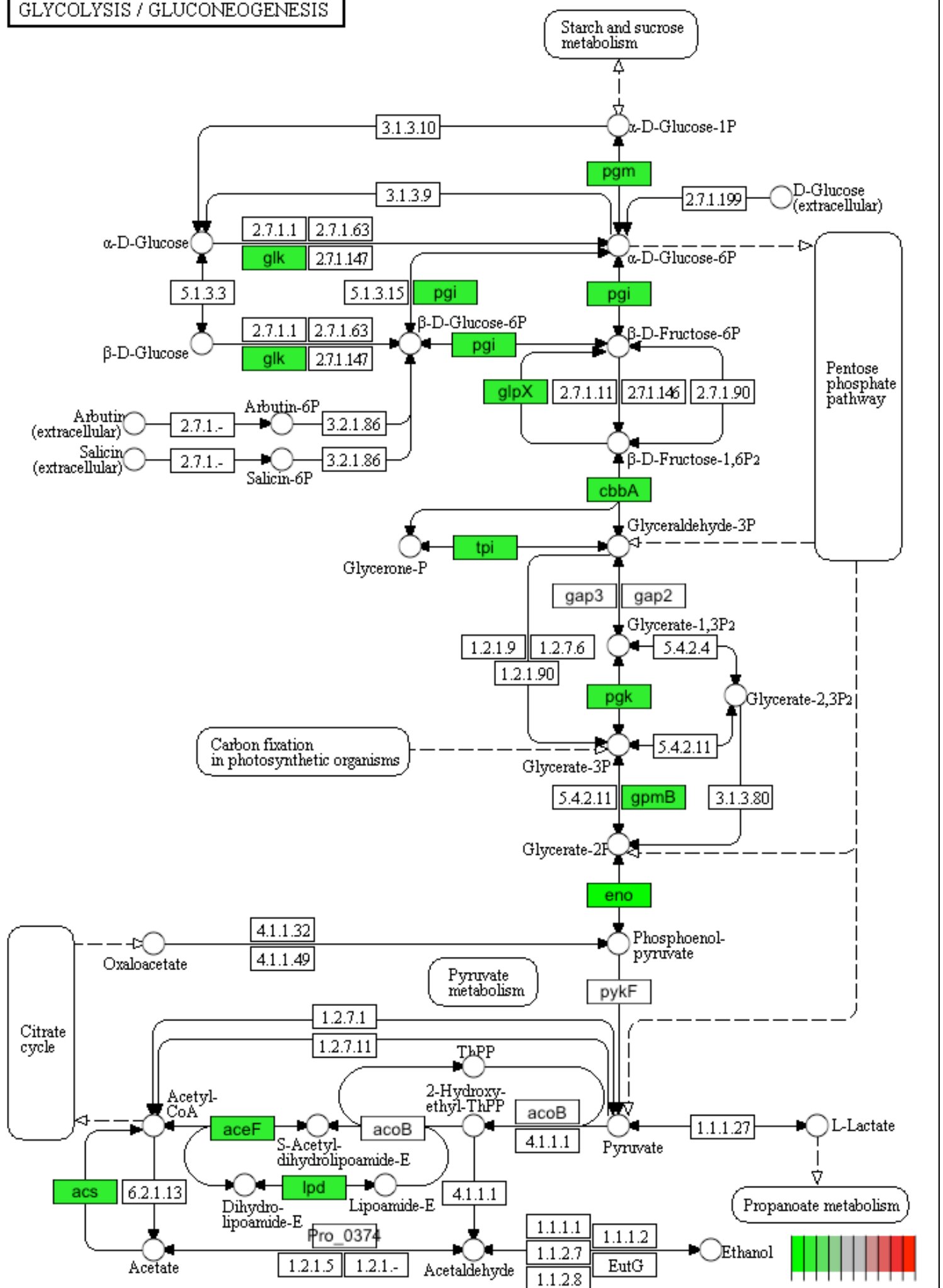

Supplement: FIG S3 [file sys003172107sf3.pdf]

Protein abundance (intensity)

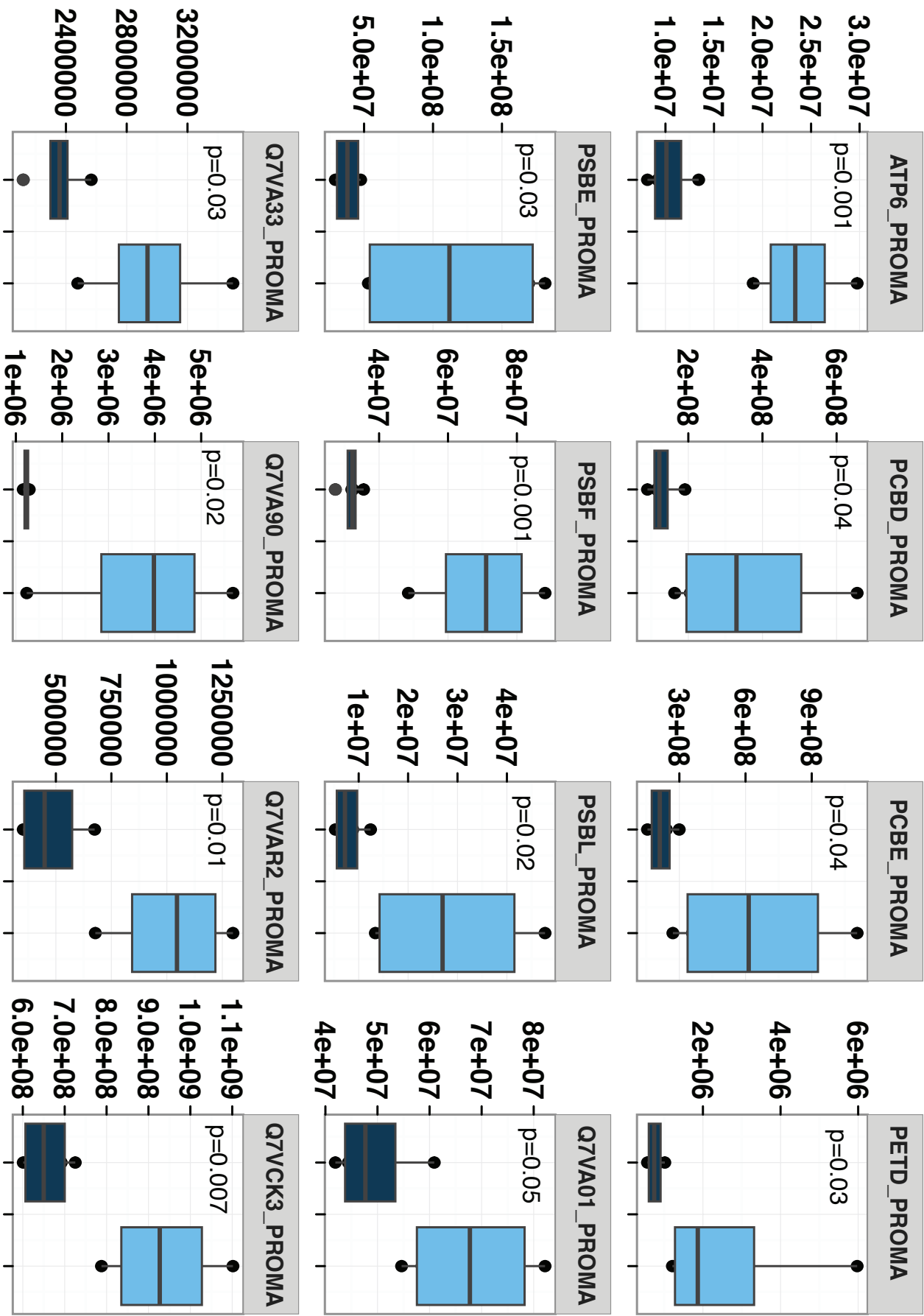

Control

Azaserine

Supplement: FIG S4 [file sys003172107sf4.pdf]

**A**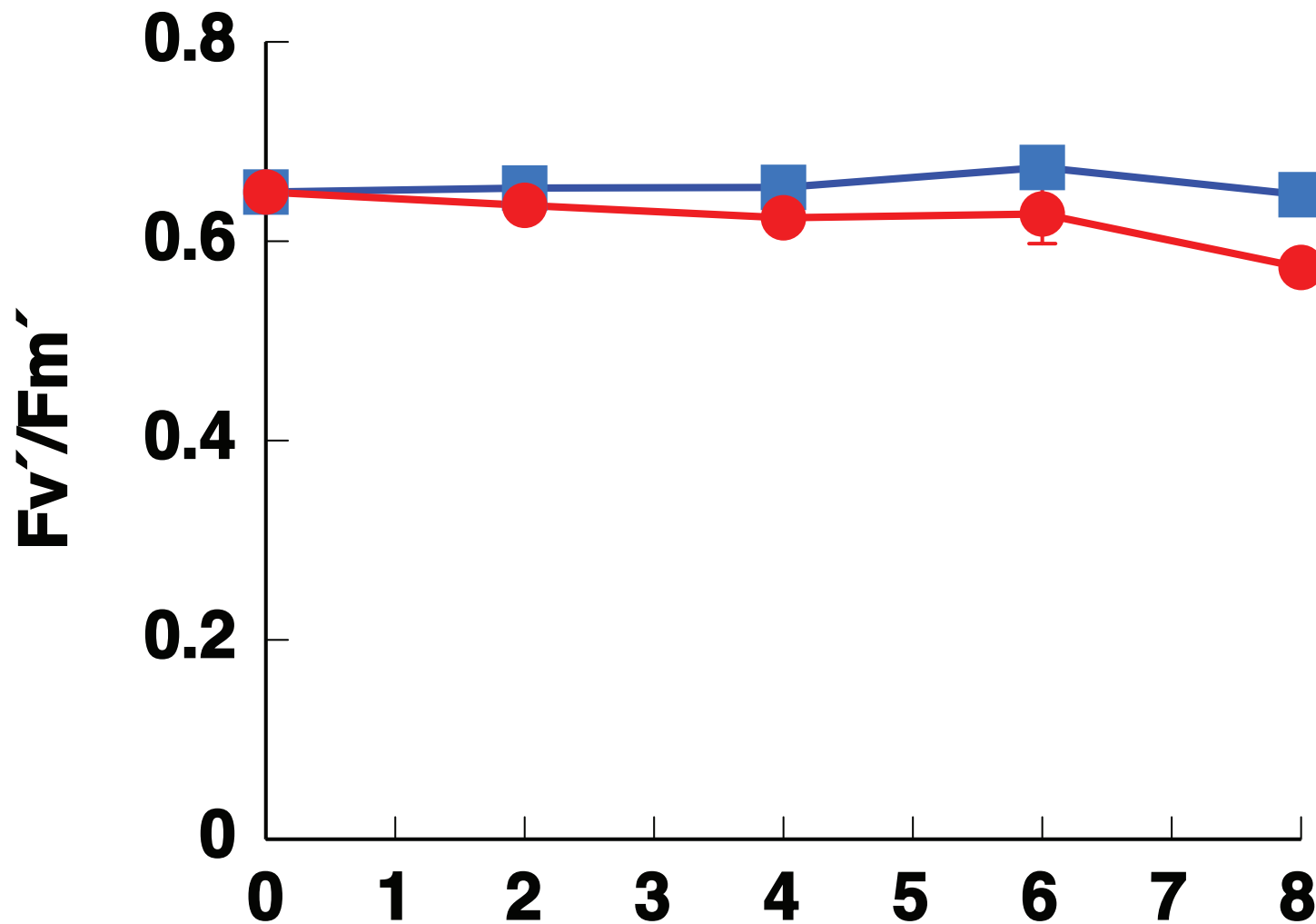**B**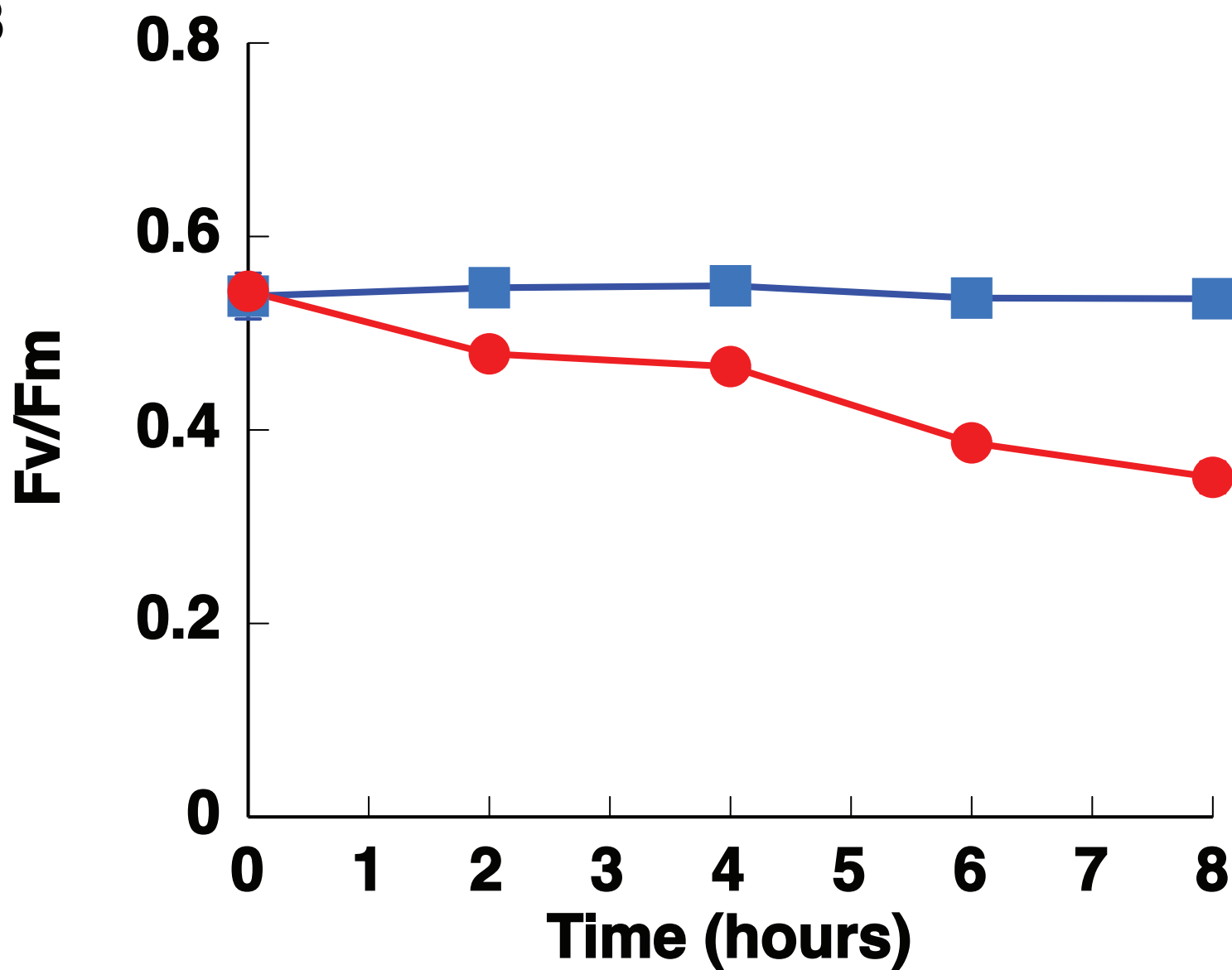

Supplement: FIG S5 [file sys003172107sf5.pdf]
