# Supplementary material for: Quantitative Proteomics Shows Extensive Remodeling Induced by Nitrogen Limitation in Prochlorococcus marinus SS120
Source: mSystems. 2017 May 30;2(3):e00008-17. doi: 10.1128/mSystems.00008-17 (PMC5451487; doi:10.1128/mSystems.00008-17)
Supplement: FIG S2 [file sys003172107sf2.pdf]

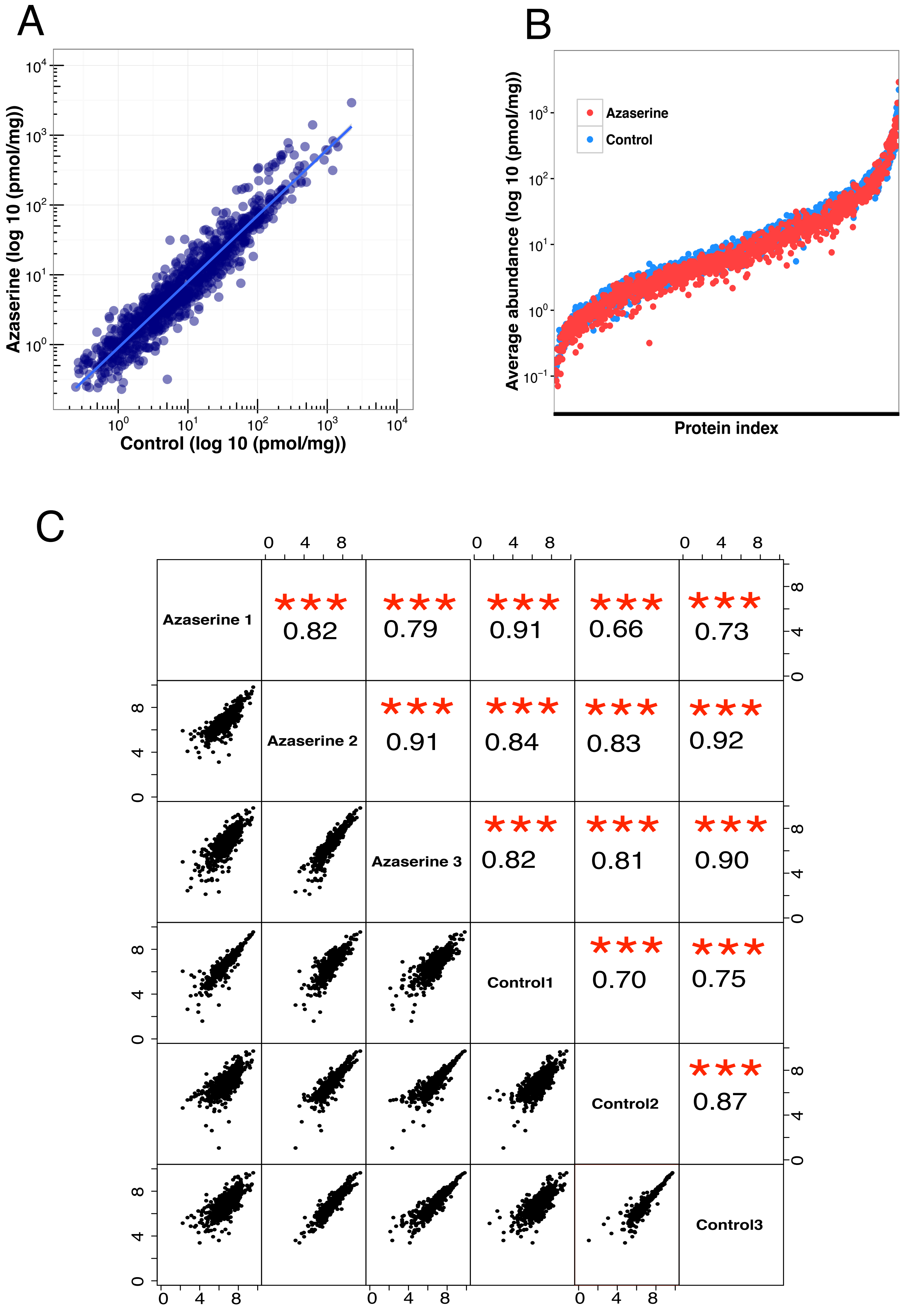
A
B
C
Azaserine (log 10 (pmol/mg))
Control (log 10 (pmol/mg))
Average abundance (log 10 (pmol/mg))
Protein index
Azaserine
Control
Azaserine 1
Azaserine 2
Azaserine 3
Control1
Control2
Control3
0.82
0.79
0.91
0.66
0.73
0.91
0.84
0.83
0.92
0.82
0.81
0.90
0.70
0.75
0.87
