# Supplementary material for: Quantitative Proteomics Shows Extensive Remodeling Induced by Nitrogen Limitation in Prochlorococcus marinus SS120
Source: mSystems. 2017 May 30;2(3):e00008-17. doi: 10.1128/mSystems.00008-17 (PMC5451487; doi:10.1128/mSystems.00008-17)
Supplement: TEXT S1 [file sys003172107s10.docx]

**Estimation of ribosome concentration in *Prochlorococcus* cells**

We used *Prochlorococcus* cultures with a cell concentration of 3 x 10^7^ cells/ml. Our cell extracts had a protein concentration of 3.2 mg protein/ml cell extract. Since we obtained a volume of 0.5 mL of cell extract from 1 L of *Prochlorococcus* cultures, we had a protein concentration of 1.6 mg of total protein in 1 L of culture, or 0.0016 mg per ml of culture.

Thus we have 0.0016 mg of protein in 30.000.000 cells; so 1 mg of total protein corresponds to 1.875 x 10^10^ cells.

The average concentration of ribosomal proteins in our control samples is 122.16 pmoles/mg of protein. Assuming there is 1 ribosomal protein per ribosome, this means 122.16 x 10^-12^ moles ribosomes x 6.023 x 10^23^ ribosomes/mol = 7.36 x 10^13^ ribosomes in 1 mg of total protein. Hence, we have 7.36 x 10^23^ ribosomes in 1.875 x 10^10^ cells, which corresponds to **3,924 ribosomes per cell in control samples**.

The average concentration of ribosomal proteins in our azaserine-treated samples is 52.34 pmoles/mg protein. By doing the same calculation described above, we have 52.34 x 10^-12^ moles ribosomes x 6.023 x 10^23^ ribosomes/mol = 3.15 x 10^13^ ribosomes in 1 mg of total protein. Hence we have 3.15 x 10^13^ ribosomes in 1.875 x 10^10^ cells, which corresponds to **1,681 ribosomes per cell in azaserine-treated samples**.
